# The Emerging Role of Senolysis in Atherosclerosis

**DOI:** 10.3390/medicina61122137

**Published:** 2025-11-29

**Authors:** Sylvia Vagena, Giorgos Theocharous, Alexios Theodorou, Pavlos Pantelis, Miltiadis Gravanis, Linnea Tscheuschner, Andreas Theodorou, George Galyfos, Frangiska Sigala, Nefeli Lagopati, Athanassios Kotsinas

**Affiliations:** 1 Department of Vascular Surgery, 1st Propaedeutic Department of Surgery, Hippocratio General Hospital, National and Kapodistrian University of Athens (NKUA), 11527 Athens, Greece; 2 Molecular Carcinogenesis Group, Department of Histology and Embryology, Medical School, National and Kapodistrian University of Athens (NKUA), 11527 Athens, Greece; 3 Department of Interventional Radiology, General Hospital of Athens, 11527 Athens, Greece; 4 Laboratory of Biology, Department of Basic Medical Sciences, Medical School, National and Kapodistrian University of Athens (NKUA), 11527 Athens, Greece; 5 Biomedical Research Foundation, Academy of Athens, 11527 Athens, Greece

**Keywords:** cellular senescence, atherosclerosis, senolytics, vascular damage, chronic disease

## Abstract

Atherosclerosis, a major contributor to vascular damage and plaque formation, is brought on by cellular senescence and chronic inflammation. A crucial matter that emerges is the classification of the disease in order to understand the pathogenic mechanisms before treatment. Given that oxidative stress, DNA damage, and inflammation contribute to cellular senescence, an increase in pro-inflammatory factors is detected in the atherosclerotic plaque, which exacerbates its instability while impeding vascular repair. This study emphasizes the importance of pathways such as Nrf2, ICAM-1, and p38 MAPK/p16^INK4A^ in the development of atherosclerosis. It also underscores the potential of senescence-targeting interventions to complement the conventional treatments for atherosclerosis. The study promotes using senolytic approaches that may serve as effective adjuncts to conventional pharmacological treatments for atherosclerosis. Particularly, quercetin, a flavonoid, demonstrates a potential action as senolytic agent by mitigating macrophage senescence, improving lipid profiles, and reducing plaque size of up to 56% in experimental models. This review article advocates for integrating senolytic approaches, including nutraceuticals like quercetin and combination therapies, to improve cardiovascular health and age-related vascular disorders.

## 1. Introduction

Atherosclerosis is the most common underlying cause of coronary, carotid, and peripheral arterial disease. While atherosclerosis in isolation rarely leads to fatal outcomes, the superimposition of thrombosis following the rupture or erosion of an atherosclerotic plaque represents the primary pathophysiological mechanism driving major clinical events (e.g., acute coronary syndromes and cerebrovascular accidents) [1,2]. The plaque formation in the vascular intima is characterized by persistent vascular inflammation, which is provoked by both standard and non-traditional risk factors [3,4]. The first definition of atherosclerosis described it as a cholesterol buildup produced by the retention of lipoproteins, particularly low-density lipoprotein (LDL), in the artery intima [5]. When LDL is picked up by a scavenger receptor, it induces an ongoing infiltration of immune cells into the atherosclerotic plaque [6,7]. Later, Russell Ross in 1999 demonstrated that atherosclerosis is an inflammatory disease, citing evidence that circulating monocytes are present in the developing fatty streak. The precise antigens that are responsible for triggering the inflammatory cascade in atherosclerosis remain incompletely characterized, with their identification currently emerging through ongoing investigative efforts [8]. Clinical trials, clonal lineage tracing, and genome-wide association have revealed that the mechanisms of innate and adaptive immune system can either stimulate or inhibit atherosclerosis [9].

An inflammatory response is triggered due to the accumulation of LDL particles in the subendothelial space, in which oxidized LDL (oxLDL) binds to pattern recognition receptors (PRRs) and Toll-like receptors (TLRs), resulting in the activation of downstream pro-inflammatory signaling pathways [10,11]. Through scavenger receptor-mediated uptake, macrophages internalize oxLDL, resulting in foam cell formation and ongoing local inflammation [12,13]. The secretion of pro-inflammatory cytokines, chemokines, and lipid mediators is stimulated by cholesterol loading in these macrophages, facilitating the recruitment of additional myeloid cells and perpetuating plaque development [14,15]. This persistent low-grade inflammatory milieu, which includes an autoimmune component, makes a significant contribution to lesion progression and destabilization [16].

Although inflammation and lipid buildup remain the traditional indicators of atherosclerosis, they do not adequately account for the instability of plaques or the frequent failure of conventional therapies to halt the progression of the disease. Cellular senescence is a distinct disease modifier that integrates oxidative stress, DNA damage, and chronic inflammation into a single pathogenic process, according to an increasing amount of evidence [17,18]. Senescence-associated secretory phenotype (SASP) from senescent cells exerts many pro-atherogenic effects, which is defined by the secretion of interleukins, matrix metalloproteinases, and growth factors that exacerbate local inflammation, promote ECM remodeling, and increase plaque vulnerability [19]. Pharmacological clearance of senescent cells with senolytic agents can reduce SASP-induced inflammation, reduce foam cell burden, and improve plaque stability in atherosclerosis models [18].

## 2. Classification of Atherosclerosis

### 2.1. Intimal Thickening and Fatty Streaks

Intimal thickening is the first vascular alteration to be observed under a microscope (AHA Type I lesion), and this is composed of layers of smooth muscle cells (SMC) and extracellular matrix (ECM). Smooth muscle cell proliferation was seen in the media prior to birth but was uncommon after birth, whereas the intima replication index was 2–5% [20].

“Intimal xanthoma” or “fatty streak” is defined by an abundance of macrophage foam cells, scattered between SMC and proteoglycan-rich intima (AHA Type II lesion) [21]. Although this entity is classified by the American Heart Association as the initial lesion of atherosclerosis, various studies propose that this lesion is reversible [21,22]. The study of Pathobiologic Determinants of Atherosclerosis in Youth (PDAY) indicates that as people age (15–34 years), lesions in the mid-right coronary arteries, abdomen ventral aorta, and the thoracic aorta may regress [23]. Under pro-atherogenic circumstances, SMCs show early indications of stress-induced premature senescence (SIPS), exposing the intima to maladaptive remodeling even though senescence is minimal at this point [24].

### 2.2. Pathologic Intimal Thickening

Pathologic intimal thickening (PIT, AHA Type III lesion) is the first lesion to progress. With an underlying lipid pool, this lesion is primarily composed of layers of SMCs grouped towards the lumen in a proteoglycan-collagen matrix. The latter consists of an acellular area with lipid insulation that is abundant in hyaluronan and proteoglycans [25]. In the lipid pool, low-density lipoproteins attach to ECM proteoglycans and are prone to aggregation [26,27]. Furthermore, structural changes in proteoglycan glycosaminoglycan chains improve the binding and retention of atherogenic lipoproteins [28].

Another distinct characteristic that defines PIT is the variable presence of macrophages on the plaque’s luminal surface, outside of the lipid pool [29]. The accumulation of foamy macrophages was discovered by Nakashima et al. in coronary artery lesions which surround branch sites in the early stages of plaque progression [29]. Additionally, the degree of free cholesterol clefts within lipid pools or at their periphery varies amongst PIT lesions [29]. Finally, a characteristic of apoptotic SMCs within lipid pools is the presence of a thick basement membrane encasing the area formerly occupied by the cell, forming the basal lamina cages [30]. Microcalcification is also present within the lipid pool, most likely due to calcifying matrix vesicles [30]. At this stage, endothelial cells (EC) and senescent macrophages proliferate, leading to elevated intercellular adhesion molecule 1 (ICAM-1) expression which also compromises cell–cell communication. The ECM is further destabilized by the SASP molecules, which increases plaque progression and susceptibility [31].

### 2.3. Fibroatheroma

**Figure 1:**
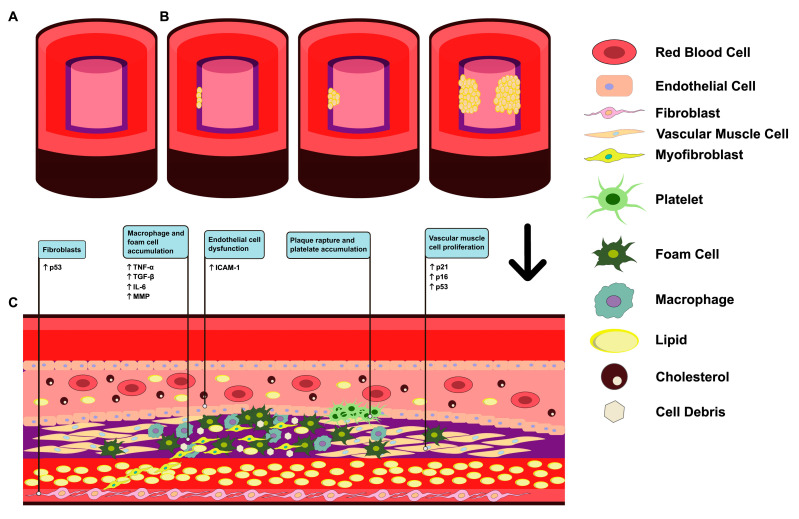
Progression of Atherosclerosis: The image illustrates the sequential stages of atherosclerotic development in arteries. (**A**) Normal Artery: A healthy artery with SMCs, unobstructed walls and absence of disease characteristics. (**B**) Fatty Streaks Stages: The sequential stages involved in the development of fatty streaks (**C**) Schematic illustration of an atherosclerotic plaque formed in the artery intima. This schema also depicts the biomarkers that characterize the distinct senescent cell populations involved in the formation of this plaque.

The acellular necrotic core of fibroatheromas (AHA Type IV lesions) is distinct from the lipid pool regions of PIT since it is made up of cellular debris and lacks matrix. The necrotic core is surrounded by a thick, fibrous cap made of SMCs embedded in a proteoglycan-collagen matrix [25]. Notably, the majority of macrophages within necrotic core regions exhibit characteristics associated with apoptotic cell death; however, emerging evidence suggests that autophagic mechanisms may also contribute to their functional state [32]. Another feature that defines the late necrotic core is the presence of free cholesterol, which is strongly linked to macrophage apoptosis [32,33]. A thick “fibrous cap” composed mainly of type I and III collagen, proteoglycans, and scattered SMCs surrounding the necrotic core [32]. The lesion’s integrity depends on the fibrous cap (Figure 1), which is prone to weakening before rupturing [32]. Apoptotic and senescent macrophages are abundant in the necrotic core of fibroatheromas. Necrotic core expansion is made worse by the persistent senescence of vascular SMCs and the impaired efferocytosis of senescent foam cells. By breaking down collagen and boosting matrix metalloproteinase activity, SASP molecules weaken the fibrous cap and increase the chance of plaque rupture [33].

## 3. Senescence

Despite that cellular senescence is characterized by various common mechanisms and features with aging, it constitutes a different phenomenon [34]. Particularly, senescence is an essential component of the aging process [35]. Cellular senescence is an intricate and dynamic phenomenon, influenced by a variety of factors such as cell type, tissue microenvironment, organismal species, and triggering stimulus. Because of its multifaced nature, a variety of approaches have been developed for accurately identifying and studying senescent cells in various contexts.

**Figure 2:**
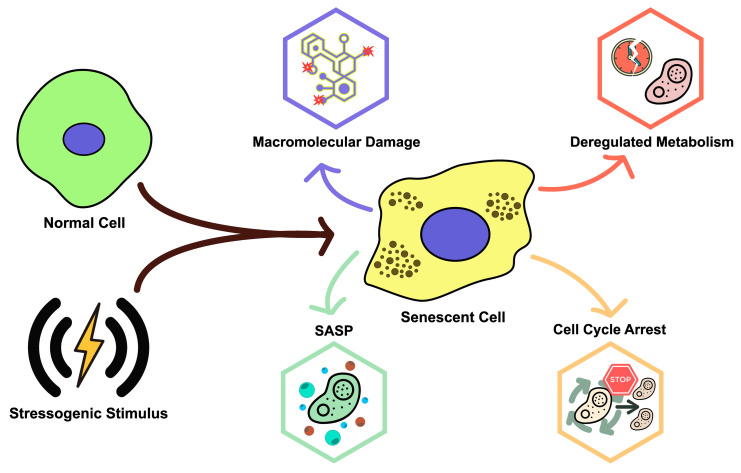
Hallmarks of senescence phenotype.

Cellular senescence occurs when cells go into an irreversible state of proliferative arrest, typically in reaction to numerous stressors. Its distinct morphological and molecular features set it apart from other non-dividing cell states (e.g., quiescence) [36]. The four main defining hallmarks of senescent cells include: (i) a stable and generally irreversible cell-cycle arrest, (ii) the SASP, (iii) macromolecular damage, and (iv) altered metabolism (Figure 2) [36,37,38]. Different types of cellular senescence have been observed, and these can be categorized according to the senescence-inducing stimuli. As research progressed over the last several decades, it became clear that Hayflick and Moorehead’s pioneering work revealed only a fraction of the true scope of cellular senescence [39]. Current research shows that cellular senescence is caused by multiple inducers except the telomere damage. Numerous telomere-independent events can cause cells to enter this state, known as SIPS. Oncogene-Induced Senescence (OIS) is caused by the incorrect activation of oncogenes such as RAS, BRAF, CDC6, AKT, CYCLIN E, and E2F1. Aside from oncogene activation, SIPS can be induced by a variety of stressors, including genotoxic damage, oxidative imbalance, chronic inflammation, radiation exposure, and various drug treatments. (Figure 2). The latter, in particular, gives rise to a distinct subtype known as Therapy-Induced Senescence (TIS) [40].

Cellular senescence has a major impact on several age-related diseases, such as metabolic syndrome, cancer, and neurodegenerative diseases. By preventing the proliferation of damaged cells, it first serves as an early cancer barrier, postponing the onset of cancer [41]. However, cellular senescence has a bright and dark side. More specifically, if it is short-term, it constitutes a vital tumor-suppressive mechanism, but if the senescence state is prolonged, it is linked with the development of chronic inflammation, which serves as a foundational driver in the progression of cancer and numerous age-related diseases. Particularly, cellular senescence can provide a powerful defense against cancer by causing permanent growth arrest and triggering the DNA damage response (DDR), which effectively stops the growth of cells with oncogenic mutations. SASP, which is a hallmark of senescent cells, releases substances that strengthen growth arrest and attract immune cells to eliminate the senescent cells. However, the prolonged expression of SASP molecules promotes chronic inflammation which creates an inflammatory environment and encourages the growth of nearby premalignant or malignant cells. These opposing features highlight the intricacy of cellular senescence and the necessity of combinational treatment approaches in cancer or age-related diseases that maximize the tumor-suppressive advantages while minimizing any potential negative consequences [42].

Mitochondrial dysfunction which provokes dysregulated metabolism are two phenomena which are implicated in cellular senescence [43]. Defective mitophagy, mitochondrial ROS, mtDNA instability, disruptions in AMPK/mTOR and NAD^+^/sirtuin pathways could not only cause but also promote cellular senescence [44]. These metabolic dysregulations are characterized by increased SASP secretion, resulting in chronic inflammation. The latest therapeutic options for targeting these pathways include metformin, mTOR inhibitors, NAD^+^ boosters, mitophagy activators, and mitochondria-targeted antioxidants [45,46].

A crucial connection between clinical atherosclerosis and cellular senescence is SASP [19]. Proteolytic enzymes MMP-2 and MMP-9, chemokines MCP-1, and inflammatory mediators like cytokines IL-6, IL-1β, and TNF-α are released by vascular smooth muscle cells, senescent ECs, and macrophages. By breaking down collagen and elastin, these secreted factors actively alter the plaque microenvironment, weakening the fibrous cap, increasing the necrotic core through poor apoptotic debris efferocytosis, and sustaining chronic vascular inflammation [47,48]. Additionally, the upregulation of adhesion molecules like ICAM-1 and VCAM-1 brought on by SASP encourages the recruitment of leukocytes over time and leads to endothelial dysfunction, which is clinically linked to microvascular angina and poor collateral vessel formation [19]. This knowledge offers a strong justification for using senolytic techniques to target SASP as a supplement to existing treatments, which may lower residual inflammatory risk and enhance outcomes for patients suffering from stroke and coronary artery disease.

An additional fact supporting the correlation between senescence and atherosclerosis is presented in bioinformatic studies that clarify significant transcriptional and pathway alterations in senescent vascular cells during the plaque progression. Pathways implicated in SASP and inflammation like NF-κB, IL-1, chemokine activation, and cytokine-cytokine receptor interactions have shown strong enrichment with consistent upregulation of core senescence genes such as p16^INK4A^ (CDKN2A), p21^WAF1/CIP1^ (CDKN1A), GDF15, ICAM-1, MMP3 and MMP9 along with suppressing endothelial repair and vascular homeostasis programs [49]. Furthermore, AMPK and mTOR pathways which are crucial for mitochondrial homeostasis and have also been linked to cellular senescence are deregulated in plaque formation as it is demonstrated by transcriptomics signatures which delineate the impairment of PGC-1a, TFAM and other electron transport chain genes [50].

### 3.1. Senescence in Atherosclerosis

#### 3.1.1. ICAM-1esion

A growing body of research has firmly established cellular senescence as a key pathological contributor to atherosclerosis, implicating a diverse array of molecular markers and signaling pathways in its progression (Table 1 and Figure 2). One of the most robust biomarkers in atherosclerosis detection is (ICAM-1). The cell surface glycoprotein ICAM-1 is produced at low basal levels by immune, EC, and epithelial cells [7]. Its expression is upregulated in response to inflammatory stimuli [51]. A mechanism connecting ICAM-1 to inflammation and immune responses is revealed by the upregulation of ICAM-1 (CD54), which is expressed by p53, which occurs independently of the NF-kB pathway.

ICAM-1 overexpression in cellular senescence and its connection to atherosclerosis have been thoroughly examined. Senescent human fibroblasts exhibit an upregulation of ICAM-1 expression, and p53 inhibitor pifithrin-α can reverse this phenomenon. The involvement of p53 in ICAM-1 regulation is also confirmed by the fact that pifithrin-α significantly decreases ICAM-1 expression in senescent human vascular smooth muscle cells (HVTs-SM1). Of note, TNF-α stimulation increases ICAM-1 expression in both young and senescent cells via NF-kB, but ICAM-1 overexpression in senescent cells is not influenced by the NF-kB pathway because the NF-kB inhibitor BAY 11-7082 does not affect it. Of note, atherosclerotic plaques are characterized by the co-expression of ICAM-1, p53, and senescence-associated β-galactosidase (SA-β-Gal). The latter unveils the elevated levels of cellular senescence in these pathological tissues and the implication of this phenomenon on the pathophysiology of atherosclerosis. In line with this notion, normal arteries do not contain SA-β-Gal-positive cells and exhibit low levels of ICAM-1 and p53 expression since senescent cells are absent [52].

ECs ICAM-1 function is significantly influenced by the aging process [53]. Late-passage human pulmonary artery endothelial cells (HPAECs) have lower ICAM-1 expression in response to TNF-α stimulation but higher baseline levels of ICAM-1 compared to early-passage cells. Following TNF-α stimulation, senescent cells also exhibit reduced ICAM-1 molecules on the cell surface, indicating that senescence reduces the expression of CD54 (cell surface ICAM-1 receptor). Single-particle tracking in senescent cells revealed a shift in ICAM-1 mobility from directed to random motion, indicating altered receptor dynamics. Furthermore, senescent cells exhibit a weaker ICAM-1-alpha-actinin interaction, since aging significantly reduces alpha-actinin phosphorylation, which is necessary for ICAM-1 clustering. Given that, these age-related alterations in ICAM-1 clustering (ICAM-1 clustering is the aggregation of ICAM-1 receptors on the cell surface into distinct patches or domains) and dynamics may interfere with cellular communication and signal transduction, which would raise the risk of vascular diseases in the elderly, the therapeutic intervention must take these changes into consideration [54].

#### 3.1.2. BRD4

The chromosomal binding protein BRD4, which controls the transcription of senescence-associated and inflammatory genes, is required for macrophage senescence [55]. Its upregulation has been linked with atherosclerosis progression and the presence of lipopolysaccharide (LPS) [55]. LPS induces senescence in THP-1 macrophages which are characterized by elevated expression of SASP molecules and senescence biomarkers [56]. The latter results in the upregulation of BRD4 expression. By blocking BRD4 with small interfering RNA (siRNA) or inhibitors such as JQ-1 and I-BET762, it is possible to effectively stop macrophage senescence, reduce the expression SASP molecules, and decrease lipid accumulation. Furthermore, conditioned medium derived from LPS-stimulated cells can induce cellular senescence in recipient cells through the SASP’s paracrine signaling mechanisms. This phenomenon can be prevented by BRD4 inhibition [57].

In addition, BRD4 can enhance the expression of SASP molecules from the macrophages by increasing cytokine production. The accomplishment of prolonged cytokine production from the macrophages requires several mechanisms, such as the elevated presence of super-enhancers associated with inflammatory responses (e.g., H3K27ac-rich loci) and NF-κB co-activation [58]. These mechanisms connect metabolic stress and cytosolic DNA sensing with persistent SASP transcription via cGAS-STING signaling, mitochondrial ROS, and NLRP3 inflammasome activity [59]. In parallel, BRD4 influences macrophage lipid handling and foam-cell fate by regulating LXR-driven cholesterol efflux pathways (ABCA1/ABCG1) and genes involved in lysosomal and autophagic flux, thus coupling inflammation with impaired lipid clearance [60]. Furthermore, BRD4 activity contributes to necrotic core expansion and plaque stability by regulating efferocytosis and matrix remodeling, implying that modulation could help restore the balance between repair and degradation processes within lesions [61]. Notably, pharmacologic strategies that improve BET targeting, such as BD2-selective inhibitors or proteolysis-targeting degraders, appear to reduce inflammatory transcription while reducing global transcriptional repression, paving the way for combination regimens with senolytics or metabolic modulators [62]. Together, these findings place BRD4 not only as a switch for SASP maintenance, but also as an epigenetic hub that integrates danger sensing, metabolism, and tissue remodeling in atherosclerotic macrophages, providing a rational framework for multi-modal intervention.

#### 3.1.3. Nicotine

Increased senescence levels and early atherosclerosis in smokers are indicators that nicotine speeds up vascular aging [63]. Nicotine increases inflammatory markers and ECM components by inducing neointima formation and vascular remodeling via the ERK and Egr-1 pathways [64]. Nicotine and cigarette smoke extract (CSE) have a major effect on VSMCs [65]. CSE reduces histone acetylation and increases the repressive histone mark H3K27me3 by epigenetically binding HDAC2 to differentiation marker promoters [66,67]. In VSMCs, nicotine causes a phenotypic shift that increases phosphorylation of ERK and p38MAPK while decreasing contractile markers [68]. Nicotinic acetylcholine receptors (nAchRs) facilitate this switch by increasing intracellular calcium [68]. Through the ERK, STAT, and p38MAPK pathways, nicotine and angiotensin II (Ang II) work together to promote VSMC migration and proliferation [69]. The upregulation of NADPH oxidase 1 (Nox1) as well as nicotine increase oxidative stress, which results in inflammation and the production of Reactive Oxygen Species (ROS) [70,71].

#### 3.1.4. T.S., DDR and NADPH and NOTCH

In addition to these effects on VSMCs, nicotine has distinct effects along the endothelial axis. Low-dose nicotine stimulates proliferation and colony formation in circulating endothelial progenitor cells (EPCs) [72,73]. This is accomplished by delaying replicative senescence via nAChR-triggered PI3K-Akt activation of telomerase, an effect that is largely NO-independent and negated by wortmannin/LY294002 or mecamylamine [72]. By suppressing NF-κB/p38/ERK signaling and restoring multiple SASP outputs, colchicine attenuates the complex tobacco smoke mixtures that cause endothelial senescence through oxidative DNA damage, Lamin B1 loss, p53/p21-mediated growth arrest, and an SASP program (e.g., TNF-α, IL-6, MCP-1, ICAM-1, MMP-2/-11) that is controlled by NF-κB and MAPKs (p38, ERK) [74]. Taken together, while nicotine may temporarily enhance endothelial repair through EPCs, ongoing smoke-induced oxidative stress and pro-senescent signaling promote endothelial dysfunction and inflammatory remodeling that accelerate atherogenesis. This imbalance can be partly restored at the endothelial level by microtubule-targeting anti-inflammatories such as colchicine.

Both stress-induced and telomere-dependent senescence can occur in VSMCs and endothelial cells [75]. Cell division causes telomeres to shorten, which results in cell cycle arrest and replicative senescence [76]. Environmental factors such as ROS and radiation cause stress-induced senescence, which triggers the DDR through ATM kinase, p53, and p21^waf1/cip1^ and results in cell cycle arrest. SA-β-gal, GL13, GLF16, p21^WAF1/CIP1^, p53, and p16^INK4A^ expression are characteristics of both types [77,78,79]. Pro-inflammatory cytokines (IL-6), growth factors (TGF-β), proteases (MMPs), ECM components are all part of the robust secretome known as SASP that senescent cells secrete [80,81]. NADPH oxidases, particularly Nox1, play a critical role in senescence and the generation of ROS [82]. While Nox4 exhibits differential effects, with downregulation causing senescence in VSMCs but inhibiting it in ECs, Nox1 stimulates ROS generation and senescence [83]. Notch1 signaling affects inflammation and induces senescence in a paracrine way by regulating SASP composition and balancing TGF-β-induced and C/EBPβ-associated secretomes [84,85].

**Table 1:** Implication of senescence in different pathways and their connection to atherosclerosis.

Pathway	Mechanism	Markers/Key Proteins	Connection to Atherosclerosis	References
Section 3.1.1 ICAM-1 Regulation via p53	p53 upregulates ICAM-1 independently of NF-κB. Senescent cells overexpress ICAM-1. Pifithrin-α reverses ICAM-1 overexpression in senescent cells.	ICAM-1, p53, SA-β-Gal	ICAM-1, p53, and SA-β-Gal are co-expressed in atherosclerotic plaques. Altered ICAM-1 dynamics in senescence impairs signal transduction and increases vascular disease risk.	[51,52,54]
Section 3.1.2 Macrophage Senescence via BRD4	LPS-induced macrophage senescence upregulates BRD4, SASP, and inflammatory markers. BRD4 inhibition decreases SASP expression and lipid accumulation.	BRD4, SASP, SA-β-Gal	BRD4 promotes macrophage senescence and SASP expression, accelerating atherosclerosis. Inhibiting BRD4 offers therapeutic potential.	[55,56,57]
Section 3.1.2 LPS-Induced Senescence	LPS activates p53, p21^WAF1/CIP1^, p16^INK4A^ and inflammatory factors, causing senescence. Different cell types exhibit SA-β-Gal activity, lysosomal content increase, and telomere shortening.	SA-β-Gal, p53, p21^WAF1/CIP1^, p16^INK4A^	LPS exposure elevates senescence markers in various cells, contributing to inflammation and vascular damage. Enhanced ICAM-1 and NF-κB activity are observed in senescent endothelial cells.	[86,87,88]
Section 3.1.3 Nicotine-Induced Senescence	Nicotine induces vascular remodeling and senescence via ERK, p38MAPK, and oxidative stress pathways. Alters VSMC phenotype and enhances ROS production.	ERK, p38MAPK, Nox1, nAchRs, SA-β-Gal	Nicotine accelerates vascular aging, promotes inflammation, and increases atherosclerosis risk. Synergizes with Ang II to enhance VSMC proliferation and migration.	[63,64,68]
Section 3.1.4 Telomere Shortening and DNA Damage Response (DDR)	Telomere attrition and ROS-induced stress activate p53-p21 pathway, leading to cell cycle arrest and senescence.	p53, p21^WAF1/CIP1^, p16^INK4A^, SA-β-Gal	Senescence-associated secretory phenotype (SASP) exacerbates inflammation and tissue damage in atherosclerosis.	[76,77]
Section 3.1.4 NADPH Oxidase-Mediated ROS Production	Nox1 generates ROS, driving senescence. Nox4 has cell type-specific effects (promotes EC health but induces senescence in VSMCs).	Nox1, Nox4, ROS, SASP	ROS-mediated senescence promotes inflammation and atherosclerotic plaque formation.	[82,83]
Section 3.1.4 Notch1 Signaling and SASP Modulation	Notch1 regulates SASP composition, balancing TGF-β and C/EBPβ-driven secretomes.	Notch1, TGF-β, C/EBPβ	Modulates inflammation and senescence in vascular cells, influencing plaque stability.	[84]

## 4. Therapeutic Approaches

Senotherapeutic approaches are divided into two categories. Senomorphics, which are also known as senostatics, and senolytics. On the one hand, senomorphics aim to mitigate the negative effects of senescent cells by suppressing the production of SASP molecules and reduce chronic inflammation. The aforementioned effects are reversible upon cessation of treatment [89]. On the other hand, the purpose of senolytics refers to the elimination of senescent cells. All in all, senolytics exert cytotoxic effects in senescent cells, whereas senostatics suppress their harmful phenotypes without eliminating them.

Senolytic treatments represent a novel therapeutic strategy in the fight against atherosclerosis by specifically targeting and eliminating senescent cells, which accumulate in vascular tissues during aging and disease. By clearing these dysfunctional cells, senolytic agents have the potential to reduce vascular inflammation, improve endothelial function, and stabilize atherosclerotic plaques, offering a promising adjunct or alternative to traditional lipid-lowering therapies (Table 2). These therapeutic approaches may serve as effective adjuncts to conventional pharmacological treatments for atherosclerosis.

### 4.1. Fisetin

The flavonoid fisetin was studied for its impact on atherosclerosis in mice with an apoE^−^/^−^ genotype. In this scientific work, mice were fed a high-fat diet and were treated with fisetin or atorvastatin, two common atherosclerosis medications [90]. Fisetin was shown to dramatically reduce atherosclerotic plaques, enhance lipid profiles, and minimize oxidative stress. Furthermore, fisetin mechanistically corrects cholesterol handling since it activates the hepatic FXR signaling, upregulates of bile-acid–synthetic/secretory machinery (e.g., CYP7A1/CYP8B1 and BSEP), and increases enterocytic ABCG5/ABCG8. These changes coincide with decreased hepatic steatosis, inflammation, and oxidative stress in HFD-fed apoE^−^/^−^mic mice [91]. In addition to these systemic effects, fisetin suppresses senescence markers (p53, p21, p16) and lowers oxidative damage (↑SOD, ↓MDA) by downregulating PCSK9 and LOX-1 in the aorta and reducing vessel-wall atherogenesis [90]. Conspicuously, fisetin and atorvastatin, a statin (HMG-CoA reductase inhibitor) used to lower LDL cholesterol, both significantly reduced aortic plaque burden and lipid accumulation in the same apoE^−^/^−^ model [90]. This highlights the potential of fisetin as an adjunct or substitute for conventional lipid-lowering therapy. Beyond lipid metabolism, fisetin has a multi-target rationale for plaque stabilization by reducing endothelial and macrophage inflammation (NF-κB/MAPK inhibition; decreased MCP-1, TNF-α, COX-2, and MMP-9), increasing cytoprotective antioxidant pathways (Nrf2-HO-1), and modulating macrophage autophagy/senescence programs (PI3K–AKT–mTOR; CKIP-1/REGγ) [92]. Thus, these findings pave the way for fisetin’s potential use as a treatment for atherosclerosis [92].

Fisetin, while frequently referred to as a “natural senolytic,” has several significant limitations that degrade its translational potential. Fisetin exhibits extremely low oral bioavailability, characterized by rapid metabolism and a short half-life, indicating that the senolytic effects observed in animal models generally require doses substantially higher than those considered safe or achievable in humans [93,94]. Its senolytic activity is also inconsistent and highly cell-type specific, frequently acting as a senomorphic rather than a true senolytic, and there is no standardized dosing regimen or validated treatment schedule [93,95,96]. Human clinical data are limited, with only small early-phase trials and no *in vivo* studies assessing vascular aging, atherosclerosis, or senescence biomarkers. Higher doses raise safety concerns, such as hepatotoxicity, anticoagulant effects, CYP-mediated drug–drug interactions, and unknown long-term effects on immunity, renal function, or reproductive health [97,98,99]. Furthermore, fisetin’s broad polypharmacology allows for potential off-target effects, and commercially available supplements vary greatly in purity and potency, with no GMP-grade clinical formulation currently available [93,100].

### 4.2. Dasatinib

Dasatinib, a BCR-ABL1 tyrosine kinase inhibitor primarily used in oncology, has several complex effects on vascular health [101]. Even though its clinical use has been linked to adverse vascular outcomes, recent studies suggest that it may have unexpected potential in controlling cholesterol metabolism and the onset of atherosclerosis. Dasatinib has been demonstrated to suppress cholesterol uptake in hypercholesterolemic mice, thereby blunting foam-cell formation and reducing the area of atherosclerotic lesions. This is consistent with a decrease in lipid accumulation in the arterial wall [102]. Beyond lipid handling, dasatinib is a senolytic (often in combination with quercetin) that uses senescent cells’ dependence on pro-survival kinase networks to selectively eliminate senescent vascular cells and reduce inflammation caused by SASP [103]. Dasatinib does this by utilizing Eph receptor signaling for Dasatinib and PI3K/AKT modules for flavonoid partners [104]. Translational reviews in cardiovascular settings highlight promising preclinical signals and early human data with Dasatinib-based regimens (e.g., D+Q showing functional gains in fibrotic disease and trends towards reduced circulating SASP factors), while also emphasizing intermittent dosing strategies and cautioning against class-specific toxicities such as cytopenia and pleural effusions prior to widespread application to atherosclerosis [105]. Understanding the molecular underpinnings of these disparate effects is necessary to explore their potential in cardiovascular applications. Interestingly, recent research on dasatinib-based senolytic formulations, such as micelle-encapsulated derivatives, indicates that targeted delivery to senescent vascular cells may reduce systemic toxicity while modulating atherosclerotic lesion development. These findings emphasize the dual relevance of dasatinib in both lipid regulation and cellular senescence pathways, opening new avenues for its repurposing in age-related vascular disease management [106].

Dasatinib has major drawbacks and safety concerns, limiting its use as a senolytic. Its activity is highly cell-type specific, and it poses several side effects such as myelosuppression, bleeding, immunosuppression, QT prolongation, cardiotoxicity, and pleural/pericardial effusions [107,108]. As a broad kinase inhibitor, it has significant off-target effects and CYP3A4-mediated drug–drug interactions, particularly with cardiovascular and anticoagulant medications [109,110]. Taken collectively, these limitations render dasatinib a precarious candidate for senolytic application beyond rigorously controlled clinical environments.

### 4.3. Quercetin

A natural flavonoid called quercetin targets the Nrf2 pathway, which controls anti-inflammatory and antioxidant responses, and has great promise for treating non-communicable diseases (NCDs) [111]. It enhances the transcription of genes (HO-1, NQO1, SOD, etc.) to reduce inflammation and oxidative stress, and it is effective against diseases such as metabolic disorders, cardiovascular diseases, and neurodegenerative diseases [112]. Through the p38MAPK/p16 pathway, quercetin also inhibits macrophage senescence, which acts in a beneficial way in atherosclerosis treatment, improves lipid profiles, lowers dyslipidaemia, and reduces plaque size by 56% in studies using ox-LDL-stimulated macrophages and ApoE knockout mice [112,113]. This is accomplished by eliminating senescent cells in the atherosclerotic plaques, restoring BMI1 expression, and inhibiting p38MAPK phosphorylation [112,113]. Despite issues with bioavailability, improvements in delivery methods could enhance its therapeutic effectiveness, making it a promising nutraceutical for cardiovascular health and atherosclerosis [114].

By activating SIRT1 and modifying the AMPK/NADPH oxidase/AKT/endothelial NO synthase signaling pathway, quercetin inhibits oxLDL-induced endothelial oxidative injuries, per Ching-Hsia Hung’s research [115]. According to a different study by Terao, dietary quercetin glycosides from plant foods support cardiovascular health by increasing bioavailability, reducing plasma LDL cholesterol, modifying gut microbiota, and having anti-inflammatory and antioxidant effects [116]. Furthermore, Kleemann et al. discovered that quercetin has anti-inflammatory, anti-proliferative, and anti-atherosclerotic effects through reducing human CRP expression, decreasing circulating risk factors such as SAA and fibrinogen, preventing atherosclerosis by 40% in ApoE*3Leiden mice, and modulating vascular inflammation and cell proliferation [117].

Quercetin exhibits several limitations that reduce its potential utility as a senolytic agent. Its activity in this context is relatively weak, variable, and markedly dependent on cell type [118,119]. It also has low bioavailability and rapid conjugation, which results in low circulating levels and unpredictable dosing [120]. Higher doses raise safety concerns, such as nephrotoxicity, CYP3A4/CYP2C9-mediated drug–drug interactions, and an increased risk of bleeding due to antiplatelet effects, while the long-term safety of repeated or chronic use remains unknown [98,120,121]. Furthermore, commercial supplements vary significantly in purity and potency, and no standardized GMP-grade formulation is available, limiting its efficacy as a senotherapeutic agent [93,122].

**Table 2:** Potential senolytic agents and their mechanisms of action as adjunctive therapies in the treatment of atherosclerosis.

Therapy/ Compound	Mechanism of Action	Key Findings	References
Section 4.1 Fisetin (Flavonoid)	Downregulates aging markers (PCSK9, LOX-1, p53, p21, p16); reduces oxidative stress	Reduces atherosclerotic plaques, improves lipid profiles, influences aging-related processes	[90]
Section 4.2 Dasatinib (BCR-ABL1 Inhibitor)	Alters cholesterol metabolism, reduces lipid uptake and lesion formation	Potentially reduces atherosclerosis but has complex vascular effects; requires further study	[101,102]
Section 4.3 Quercetin (Flavonoid)	Activates Nrf2 pathway, inhibits p38MAPK/p16 pathway, reduces senescence markers	Lowers plaque size by 56%, improves lipid profiles, reduces inflammation and oxidative stress	[111,112,113,114]

## 5. Conclusions and Future Perspectives

Life-threatening conditions like coronary artery disease and stroke are caused by atherosclerosis, a chronic inflammatory disease characterized by the presence or the concentration of cholesterol and immune cells in arterial walls. Cellular senescence, which is fueled by oxidative stress, DNA damage, and inflammation plays a crucial part in the development of atherosclerosis since it releases pro-inflammatory factors and hinders vascular repair mechanisms. SASP destabilizes atherosclerotic plaques and increases inflammation according to the latest bibliographic data. Targeting cellular senescence to manage atherosclerosis can be very promising, especially by modifying pathways like p38MAPK/p16, ICAM-1, and BRD4.

Quercetin and dasatinib have very different relationships with atherosclerosis in humans. Quercetin supplementation (500 mg/day) in post-MI patients reduced TNF-α and hs-CRP levels, increased antioxidant capacity, and improved quality of life. This aligns with its experimental actions of increasing the Bcl-2/Bax ratio and activating PI3K/Akt to limit apoptosis, oxidative stress, and inflammation, which are key processes in atherogenesis and plaque instability. Large clinical datasets of dasatinib show that overall cardiovascular ischemic event rates (≈2–4%) are not higher than expected when compared to matched external populations, indicating no clear excess atherosclerotic risk associated with the drug [123]. However, most ischaemic events on dasatinib occur in patients with pre-existing atherosclerosis or major risk factors and usually occur within the first year of treatment [124]. When taken together, quercetin may have anti-atherosclerotic and vasculoprotective effects, whereas dasatinib appears to be cardiovascularly neutral overall, but can mask or coincide with events in people with a high underlying atherosclerotic burden, emphasizing the importance of careful risk assessment in that population.

A multifaceted therapeutic strategy that integrates existing pharmacological agents with emerging senolytic drugs could offer a groundbreaking, less invasive approach to the prevention and management of atherosclerosis. By simultaneously targeting lipid metabolism, vascular inflammation, and cellular senescence, this combination therapy has the potential to not only slow disease progression but also enhance vascular repair mechanisms, ultimately reducing the long-term burden of cardiovascular disease.

## 6. Methodology of Literature Review

Systematic search and review of articles regarding atherosclerosis, cellular senescence and senolytic drugs was conducted using PubMed, Scopus, and Science Direct. The keywords used were atherosclerosis; senolytics; mitochondrial dysfunction; cellular senescence; and cardiovascular disease.

## Abbreviations

The following abbreviations are used in this manuscript:

CSE	Cigarette Smoke Extract
DDR	DNA Damage Response
EC	Endothelial Cells
ECM	Extracellular Matrix
HPAECs	Human Pulmonary Artery Endothelial Cells
HUVECs	Human Umbilical Vein Endothelial Cells
HVTs-SM1	Human Vascular Smooth Muscle Cells
ICAM-1	Intercellular Adhesion Molecule 1
LDL	Low Density Lipoprotein
LPS	Lipopolysaccharide
nAchRs	Nicotinic Acetylcholine Receptors
NCDs	Non-communicable diseases
Nox1	NADPH Oxidase 1
OIS	Oncogene-induced senescence
oxLDL	Oxidized LDL
PBMCs	Peripheral Blood Mononuclear Cells
PIT	Pathologic Intimal Thickening
PRRS	Pattern recognition receptors
ROS	Reactive Oxygen Species
SA-β-Gal	Senescence-Associated β-galactosidase
SASP	Senescence-Associated Secretory Phenotype
SIPS	Stress-induced premature senescence
siRNA	Small Interfering RNA
SMC	Smooth Muscle Cells
TIS	Therapy- induced senescence
TLRs	Toll-like receptors
